# Development of a Detailed Volumetric Finite Element Model of the Spine to Simulate Surgical Correction of Spinal Deformities

**DOI:** 10.1155/2013/931741

**Published:** 2013-08-07

**Authors:** Mark Driscoll, Jean-Marc Mac-Thiong, Hubert Labelle, Stefan Parent

**Affiliations:** Spinologics Inc., 289 Desaulniers, Saint-Lambert, QC, Canada J4P 1M8

## Abstract

A large spectrum of medical devices exists; it aims to correct deformities associated with spinal disorders. The development of a detailed volumetric finite element model of the osteoligamentous spine would serve as a valuable tool to assess, compare, and optimize spinal devices. Thus the purpose of the study was to develop and initiate validation of a detailed osteoligamentous finite element model of the spine with simulated correction from spinal instrumentation. A finite element of the spine from T1 to L5 was developed using properties and geometry from the published literature and patient data. Spinal instrumentation, consisting of segmental translation of a scoliotic spine, was emulated. Postoperative patient and relevant published data of intervertebral disc stress, screw/vertebra pullout forces, and spinal profiles was used to evaluate the models validity. Intervertebral disc and vertebral reaction stresses respected published* in vivo*, *ex vivo*, and *in silico* values. Screw/vertebra reaction forces agreed with accepted pullout threshold values. Cobb angle measurements of spinal deformity following simulated surgical instrumentation corroborated with patient data. This computational biomechanical analysis validated a detailed volumetric spine model. Future studies seek to exploit the model to explore the performance of corrective spinal devices.

## 1. Introduction

Computational analyses, such as finite element modeling, have benefited from the ever-growing performance of computers allowing them to reliably simulate the complex biomechanical behavior of the musculoskeletal system. Moreover, this platform allows for a cost and time effective manner in which head-to-head device comparison may be objectively conducted. However, this mathematically complex form of analysis must be performed and interpreted with caution.

With regard to modeling of the spine, the last ten years have seen an influx of published models exploring spinal biomechanics. Using volumetric and rigid body models, Aubin et al. have pioneered work in the understanding of the pathomechanism of scoliosis [1–5], the mechanics of spondylolisthesis [6], the correction forces offered during scoliotic brace treatment [7–9], and the performance of next generation of minimally invasive growth modulation devices [10, 11]. Using rigid body models, Aubin et al. also simulated many times the surgical correction of spinal deformities via the introduction of rods and screw [12, 13] while others followed suit [14–16]. The aforementioned rigid body models use multilinear elastic beams and cables as a simplified spinal geometry and are at times constructed based on accurate 3D reconstructions from patient geometry [17]. A potential fallback of rigid body modeling and patient based construction is that analyses of internal stress distributions and hypothetical cases, without the support of actual patient data, cannot be easily explored. These shortcomings make optimization analyses, which explore a spectrum of hypothetical scenarios to derive reliable biomechanical insight, very cumbersome. On the opposite, detailed finite element modeling of the spine allows for more complete assessments of internal stress distribution within the model physiological tissue and instrumentation construct. Unfortunately, there is currently no available finite element model of the complete spine that can simulate the surgical correction of spinal disorders, as detailed FEM involving spinal instrumentation is limited to short segments of the spine.

The purpose of this preliminary study was to develop a parametric volumetric detailed finite element model of the osteoligamentous spine to simulate surgical corrections of common spinal pathologies which, in subsequent studies, will serve to compare, design, and optimize new spinal devices.

## 2. Material and Methods

### 2.1. Finite Element Model

A detailed custom finite element model (FEM) was coded using ANSYS APDL language (ANSYS 13.0 APDL, Canonsburg, PA). User input defines the 6 degrees of freedom and volumetric scale between subsequent adjacent vertebral bodies which, as a result of the constructive geometric overlay of the vertebrae and intervertebral disc, afford the user complete control over the end results spinal profile in all anatomical planes. The intervertebral discs were attributed to a modulus of elasticity of 8 MPa and 2 MPa [18]. The anterior longitudinal, posterior longitudinal, interspinous, ligamentum flavum, and capsular ligaments were modeled providing tensional forces defined by 23.75, 26.15, 9.8, 22.6, and 23.7 Newtons per mm of deformation, respectively, [19]. The vertebral bodies were modeled as rigid bodies. The superior surface of T1 was translationally constrained in the transverse plane while the inferior surface of L5 was allowed only coronal rotation.

**Figure 1:**
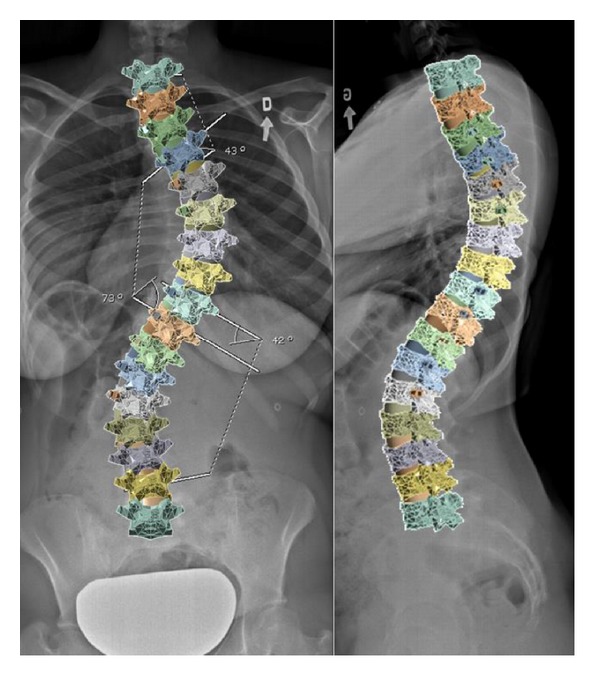
Coronal and sagittal radiographs (thoracic curve 73°, kyphosis 35°, and lordosis 37°) and superimposed FEM (thoracic curve 69°, kyphosis 32°, and lordosis 36°).

The selected patient to model had a scoliotic curve defined by a primary right thoracic Cobb angle of 73° and a proximal thoracic Cobb angle of 42° (Lenke type 2B). Lateral bending tests revealed the primary curvature to correct to 40° or by 45%. Postoperative radiographs showed the spine to correct to 20° or by 73%. The sagittal profile of the patient was defined by a pre- and postoperative thoracic kyphoses of 35° and 26° between T1 and T12, respectively, and a corresponding lumbar lordosis of 37° and 36° between L1 and L5. The model was constructed to represent the patient's spinal profile within 5° (Figure 1). Surgical correction was made by instrumenting the concave side of the curve using pedicle screws from T3–T6 and T9-L1 connected by a 5.5 mm diameter rod. A convex rod was used to stabilize the construct.

Titanium (Ti-6Al-4V, grade 5) pedicle screws and the cobalt chrome rod had a modulus of 11 and 213 GPa, respectively. Contact between the screw shaft and the intervertebral body was programmed by coupling neighbouring element/nodes. The polyaxial screw head was simulated with a restricted outwardly conic degree of freedom of 60° governing the angle at which the rod was permitted to be captured within the screw head. The 5.5 mm spinal rod was shaped with respect to the desired sagittal profile.

### 2.2. Simulation of Surgical Steps

**Figure 2:**
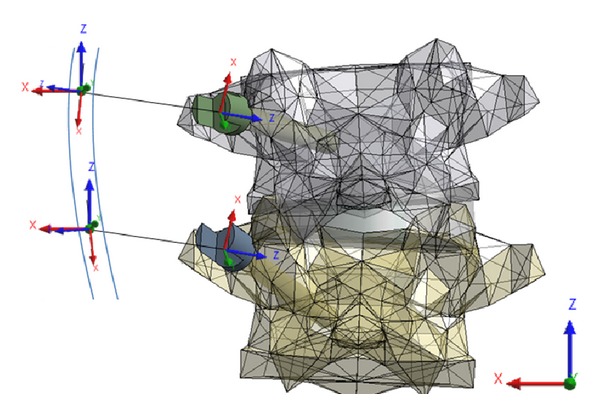
Example of artificial linkage coordinates systems between screw heads and spinal rod.

Segmental translation of the concave rod was simulated using several superimposed steps. Stabilization rod was not modeled. All bodies were assigned numerous coordinate systems centered to their geometric center of mass to control and measure movement. An artificial linkage was provided between every screw head and the spinal rod to reduce the distance between them in a controlled manner. This linkage consisted of several coordinate systems each with its own custom coded degrees of freedom (Figure 2). A first joint was fixed to the center of the screw head and allowed to translate along the *z*-axis which was aligned parallel to the second joint of the linkage located on the spinal rod. The second joint was free to rotate along its *z*- and *y*-axes and had a coordinate system located at the cross-sectional center of the rod and whose *x*-axis was tangentially aligned to the spinal rod profile. A third joint, which nested and shared origins with the second joint, was free to translate and rotate with respect to its *z*-axis which was aligned parallel to the global or axial axis of the spine. As needed, stiff bushings were introduced into the first joint to encourage convergence.

**Figure 3:**
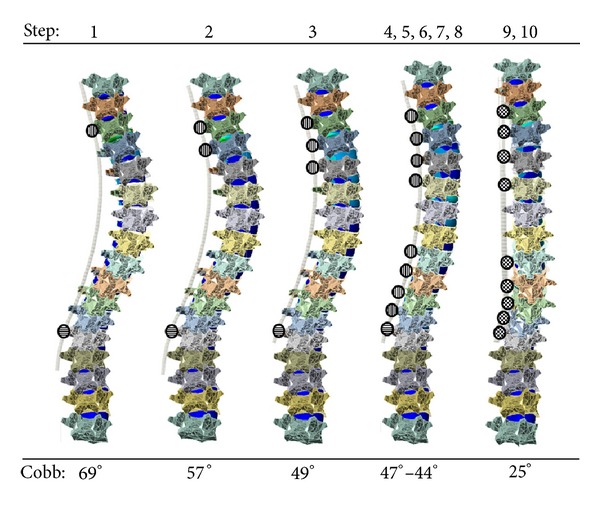
Simulated surgical steps of segmental translation and spinal rod derotation of a scoliotic spine (circles filled with longitudinal, horizontal, and checkered graphics represent translation/rotation, rotation only, and fixed degrees of freedom of rod within screw heads, resp.).

Surgical simulation began with the most cranial (T3) and caudal (L1) screws being housed with the rod and having linkages permitting axial rotation and translation (third joint *z*-axis) and only axial rotation (third joint *z*-axis, Figure 3, step 1) representing the set screw not being completely tightened. The distance between the screw head and rod was then subsequently reduced along the *z*-axis of the first joint moving from cranial (T4) to caudal (T12) (Figure 3, steps 3–8). Next, the rod was fixed in position while permitting translation and rotation in the global axial plane (third joint *z*-axis) while the most caudal screw L1 allowed only rod rotation. Finally, the rod was rotated until the predetermined spinal profile was found in the sagittal plane (Figure 3, step 9) and screw secured (Figure 3, step 10).

### 2.3. Validation

Intervertebral disc stresses were continuously monitored and reviewed for adherence to *in silico*,* ex vivo*, and* in vivo *published data. Pullout forces measured at the screw/vertebra interface were also monitored and compared to relevant prior studies using biomechanical validated models simulating corrective scoliotic surgeries and to found threshold values suggesting risk of failure [20–22]. Postoperative curvatures from patient data and predicted values of the model, in terms of spinal profiles, were also compared to evaluate the reliability of the computational reactions of the model when imposed to comparable surgical instrumentation and maneuvers as performed in practice.

## 3. Results

The final FEM of the thoracic and lumbar spine instrumented on the concave side consisted of 66 volumetric bodies, 33 contact regions, 96 tension only elements (ligaments), and 43 joints. Measures of intervertebral disc stress (von-Mises) were maintained within acceptable values as reported in Table 1. These measures represent the average stress measured in the entire disc over the course of the surgical simulation for discs not being spanned by instrumentation (i.e., between T1–T3, T6–T9, and L1–L5). Measures of stress in intervertebral discs found between instrumented vertebrae averaged at stress of 3.95 MPa over the entire simulation of the surgical procedure. No consistent correlation between peak measures of intervertebral disc stress and the simulated surgical maneuver was observed.

**Figure 4:**
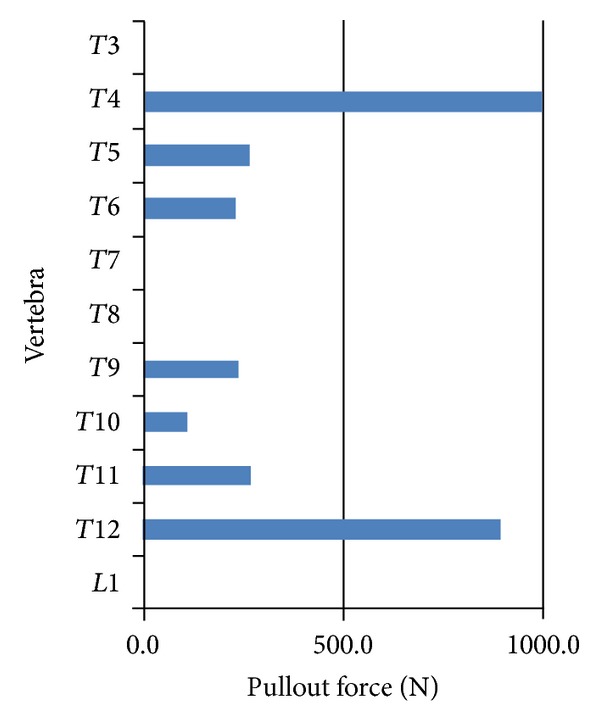
Maximum pullout forces measured during the simulation for each vertebra.

**Figure 5:**
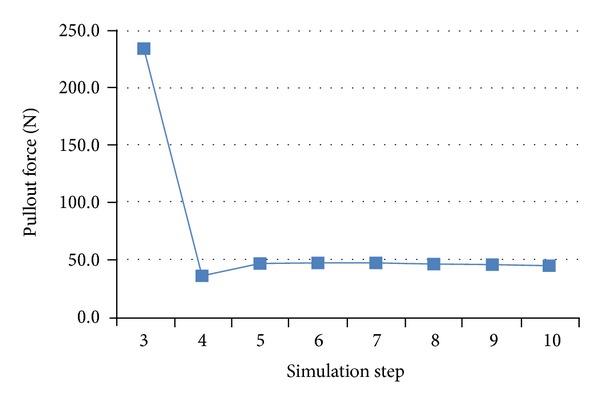
Pullout forces at T6 during simulated segmental translation of scoliotic spine model.

Pullout forces acquired at the screw/vertebra interface of all pedicle screws are reported in Figure 4. Most commonly, in five of seven screws (T3–T5, T9, and T11), the peak screw pullout force was experienced during the initial rod capture. Otherwise peak pullout was experienced during the simulated rod derotation maneuver (T10 and T12). In screws having their peak pullout during initial capture, the common force pullout versus surgical step graph resembled that of T6 exemplified in Figure 5. In this scenario, T6 pullout forces peaked when the concave screw was joined to the rod (Figures 3 and 5, step 3) and then reduced during subsequent steps during which the rod was attached to the subsequent screw heads (Figures 3 and 5, steps 4–10).

Spinal profiles adhered to patient data within the instrumented thoracic and lumbar regions of the model within the targeted 5°. The postoperative thoracic Cobb angles from patient and simulated data were 20° and 25° while the kyphoses were 26° and 28° respectively. Outside the instrumentation in the lumbar region the postoperative lordosis was measured at 36° and 30° for patient and simulated data correspondingly. To achieve this corroborative data the mechanical properties of the intervertebral disc annuli were adapted to 5 MPa. The primary thoracic Cobb angle of the model was reduced after each iteration of the simulated surgical steps as detailed in Figure 3. Steps 4–8 saw little in scoliotic reduction of the Cobb angles as the rod had little to travel to be secured to the screw heads. Maximum correction of the thoracic Cobb angle was experienced during the simulation of the rod derotation maneuver, step 9, when it reduced from 44° to 25°.

## 4. Discussion

This study utilized a custom coded parametric and volumetric model of the osteoligamentous thoracic and lumbar spine to simulate common surgical techniques used in the correction of scoliotic spines. Stresses measured in the intervertebral discs not spanning an instrumented segment are consistent with the published literature. Discs that spanned instrumented vertebrae showed an average stress higher than such a range perhaps due to local manipulation and/or compressions introduced by the simulated surgical processes.

Pullout forces measured at the screw/vertebra interface were in agreement with relevant computational models. Wang et al., in a well-performed study, explored the pullout forces using both monoaxial and a multidegree of freedom postloading screws and reported forces up to 886 Newtons [13]. Aubin et al. found pullout forces from simulated up to 1073 Newtons [12]. Complementing FEM studies of pedicle screw pullout documented between 1218 to 1892 Newtons [20]. Results of pullout forces experience in the developed model were well within these ranges suggesting that simulated reaction forces were sound. 

Coupling the simulated spine model profile to the patient data required modification to intervertebral disc material properties causing successful corroboration within the instrumented region. This is due to accurately modeled mathematical relations between the vertebrae, screws, and the spinal rod. Outside the instrumented area, the lumbar spine had less accuracy. This resides with difficulties to simulate response of the spine outside the instrumentation region which depends on secondary reactions from overhead simulated surgical maneuvers.

The model proposes a novel *in silico* platform offering a means to explore new medical devices for the treatment of spinal pathologies. A number of assumptions must be considered when seeking to extract concrete insight. The variability within the mechanical behavior of physiological tissue can hinder any models conclusions, such as those governing the response of the noninstrumented curve. Alternative studies, given the strict parameterization of the model, could easily compare, as an example, influence of utilizing different rod materials, use of other anchors (hooks and wires) and correction maneuvers such as compression, distraction, segmental derotation, and/or *in situ* bending.

This preliminary validated model of the spine allows one to monitor the intervertebral disc stresses and screw pullout forces over the course of the simulated surgery. The models purpose is to provide insight into optimal fixation techniques. Furthermore, this model may serve as a valuable tool to assist in different stages of product development such as concept validation, product optimization, *in silico *bench testing, regulatory affairs assistance, marketing support, and/or head-to-head device comparisons. 

## Figures and Tables

**Table 1:** Published *in vivo*, *ex vivo*, and *in silico* values of intervertebral disc stresses.

Author	Method	Disc Location	Disc Section	Mean stress (MPa)
Adams 1996 [23]	*Ex vivo *	L4-5	Nucleus	1.6
			Anterior	2
			Posterior	2.6
Wilke 1999 [24]	*In vivo* sanding	L4-5	Nucleus	0.5
Schultz 1982 [25]	*In vivo* sanding	L4-5	Nucleus	0.27
Nachemson 1965 [26]	*In vivo* standing	L4-5	Nucleus	0.87
Andersson 1974 [27]	Computational	L4-5	Nucleus	0.3–0.5
Meir 2007 [28]	*In vivo* lateral	Apex	Concave	0.8–0.4
			Convex	0.15
Sato 1999 [29]	*In vivo* prone	L4-5	Nucleus	0.15
Shrzypiec 2007 [30]	*Ex vivo *	C7-T1	Nucleus	1
			Anterior	1.35
			Posterior	1.1
Steffen 1998 [31]	*Ex vivo *	L3-4	Nucleus	0.8
Schroeder 2006 [32]	FEM	L4-5	Nucleus	0.6–0.85
			Anterior	0.6–1
			Posterior	0.8–1.2
*Current Study *	*FEM *	*T1–T3 *	*Average *	*0.59 *
		*T6–T9 *		*2.56 *
		*L1–L5 *		*0.81 *
